# Two decades of Neo-Marxist class analysis and health inequalities: A critical reconstruction

**DOI:** 10.1057/sth.2015.17

**Published:** 2015-08-05

**Authors:** Carles Muntaner, Edwin Ng, Haejoo Chung, Seth J Prins

**Affiliations:** a Bloomberg School of Nursing, Dalla Lana School of Public Health, University of Toronto, 155 College Street, Suite 386, Toronto, Ontario, Canada M5T 1P8; b Centre for Research on Inner City Health, Li Ka Shing Knowledge Institute, 209 Victoria Street, 3rd Floor, Toronto, Ontario, Canada M5B 1C6. E-mail: nged@smh.ca; c Department of Public Health Sciences, Korea University, Suite 365, Hana Science Building, 145 Anam-Ro, Seongbuk-Gu, Seoul, 136-713 Republic of Korea. E-mail: hpolicy@korea.ac.kr; d Department of Epidemiology, Columbia University, Mailman School of Public Health, 722 West 168th Street, Suite #720C, New York, NY 10032, USA. E-mail: sjp2154@columbia.edu

**Keywords:** social class, Neo-Marxist, mechanism, exploitation, health inequalities

## Abstract

Most population health researchers conceptualize social class as a set of attributes and material conditions of life of individuals. The empiricist tradition of ‘class as an individual attribute' equates class to an ‘observation', precluding the investigation of unobservable social mechanisms. Another consequence of this view of social class is that it cannot be conceptualized, measured, or intervened upon at the meso- or macro levels, being reduced to a personal attribute. Thus, population health disciplines marginalize rich traditions in Marxist theory whereby ‘class' is understood as a ‘hidden' social mechanism such as exploitation. Yet *Neo-Marxist social class* has been used over the last two decades in population health research as a way of understanding how health inequalities are produced. The Neo-Marxist approach views social class in terms of class relations that give persons control over productive assets and the labour power of others (property and managerial relations). We critically appraise the contribution of the Neo-Marxist approach during the last two decades and suggest realist amendments to understand class effects on the social determinants of health and health outcomes. We argue that when social class is viewed as a social causal mechanism it can inform social change to reduce health inequalities.

## Introduction

The term ‘Neo-Marxism' has been applied during the last century to a number of social science currents that expanded the work of Karl Marx with input from other intellectual developments. In particular in the 1980s emerged a distinctive school of Neo-Marxists, Analytical Marxism (Roemer, 1986) that not only integrated other social science theoretical traditions (for example, Weberian sociology, Neo-classical economics, game theory, development economics and political philosophy) but also embraced empirical class analysis (Wright and Perrone, 1977; Wright, 1985). Analytical Marxism has proved more influential than originally anticipated (Jacoby, 1989). For example, a social scientist influenced by Analytical Marxism has become influential in worldwide academic and policy circles dealing with income inequality (Piketty, 2014). An economist associated with this current has become Greece's Finance Minister and is now in charge of the negotiations with the European Union on his country's debt. The health field, in particular the sociology of health and illness (Muntaner *et al*, 2013) and social epidemiology (Galobardes *et al*, 2006) have also been a fertile ground for this kind of Neo-Marxist influence. Specifically, the theoretical and empirical research by Wright (1985, 2000) on Neo-Marxist class analysis has translated in new explanations and findings on the relation between class and health (Muntaner *et al*, 2013), a central question for the sociology of health (Hollingshead and Redlich, 1953).

The term ‘social class' is widely used in health inequalities research. Nonetheless, it remains a contested concept. Broadly speaking, three major uses of ‘social class' coexist in the health field: pragmatic, Neo-Weberian and Neo-Marxist (Muntaner *et al*, 2000). The pragmatic approach has typically focused on simple stratification indicators such as income, educational attainment, and occupational hierarchies that may, *de facto,* draw on a functionalist tradition in sociology, and even a Weberian concept of status (Muntaner *et al*, 2000). The Neo-Weberian approach typically focuses on processes of opportunity hoarding or social closure in market relations, through measures of educational credentials and occupational prestige. The Neo-Marxist approach, elaborated below, can be summarized by a focus on relations of economic production, through processes of ownership and labour, domination and exploitation. Other theories of class have also gained interest in population health research; for example, Bourdieu's framework, in which groupings of individuals in a multidimensional social space, with various forms of capital – social, economic, cultural, and symbolic – share circumstances and interests and can manifest classes (Bourdieu, 1987; Veenstra, 2007).

Although these three approaches have been subject to several empirical comparisons in sociology (Marshall *et al*, 2005) and population health (Wolfarth, 1997; Muntaner *et al*, 2003), the theoretical and empirical status of the Neo-Marxist approach has yet to be examined. As a consequence this article will focus on the merits of Neo-Marxist approaches to social class in the sociology of illness during the last two decades, approximately when Neo-Marxist constructs and measures began to be used in socio-epidemiologic journals (Muntaner *et al*, 2013). We critically review its contributions and point to new directions to better explain the resilient association between social class and health. We begin by critiquing current methods in health sociology and social epidemiology, and attendant implications for reducing health inequalities. Next, we lay out the origins of the Neo-Marxist class concept used in empirical studies of population health, its main findings, limitations and what could be a heuristic approach. A key suggestion is that a focus on social mechanisms might overcome the lack of relative explanatory power of employment relations indicators. We conclude by discussing the implications of a Neo-Marxist class approach for applied population health interventions, and identifying future directions for research and practice.

## The Individualization of Social Class

Most population health researchers conceptualize social class as a set of attributes and material conditions of life of individuals (Krieger *et al*, 1997). The empiricist tradition of ‘class as an individual attribute' equates class to an ‘observation', precluding the investigation of unobservable social mechanisms (Muntaner *et al*, 2013). Another consequence of this view of social class is that it cannot be conceptualized, measured, or intervened upon at the meso- or macro levels. Thus, population health disciplines marginalize rich traditions in Marxist theory, whereby ‘class' is understood as a ‘hidden' social mechanism such as exploitation.

The marginalization of rich traditions of class theory in favour of the mainstream, individual-attribute approach, is consistent with the biological, psychological, and quantitative reductionism that characterizes the methodological individualism of contemporary social science, and the reduction of social science to a technique, which has both reflected and affected cultural, political, and economic processes in contemporary class relations. When, consistent with this trend, social processes such as class relations are conceptualized as individual attributes, it absolves researchers from engaging with social mechanisms (of which class relations are a prototype). In population health research and theory, examples include prioritizing individual-level risk factors over population determinants of risk (that is, the risk of risk) (Rose, 1985); disqualifying as valid causal constructs any variables that are not easily manipulable in the current policy space (for example, ‘no causation without manipulation') (Hernán, 2005); prioritizing research on commodifiable, purportedly biological mechanisms for socially conditioned physical and mental health problems (Burke *et al*, 2010); and re-reifying ‘race,' for example, the re-emergence of genetic essentialism, rather than focus on the social processes of racialization and racism (Duster, 2005).

This approach to social class has real consequences. For example, think tanks in the 1980s and 1990s provided empirical justification for the Temporary Assistance for Needy Families program, legislation that ended the possibility to receive insufficient but secure aid for the poor in the United States (‘welfare reform'). The success of such research was possible by reducing social inequalities to individual stratification indicators such as education and ‘race' that individualized social outcomes (for example, income) and by omitting sociological processes from their analysis (for example, social class, racial segregation, access to education, housing and social services) (O'Connor, 2009). More recently, the report of the WHO Commission on Social Determinants of Health (WHO, 2008) explicitly avoided the social class construct. The result of this omission is that the report tells us little about the origins of economic and power inequalities, and that we all seem to be equally responsible for health inequities (Navarro, 2009).

Although, the mainstream approach to social class has dominated health sociology and social epidemiology, an alternative approach, drawing on Neo-Marxist theory, has developed in tandem. This approach holds promise – empirically, theoretically, and practically – for advancing the study of health inequalities and providing an intellectual foundation for the social change required to reduce them.

## Street Fighting Sociologists

The emergence of empirical Neo-Marxist class analysis in population health developed in the 1990s stemming from the work of sociologists such as Melvin Kohn, in the 1960s and 1970s. Drawing on the theoretical writings of European scholars such as Poulantzas (1978) and Dahrendorf (1959) who tackled the problem of power and control over production in class analysis, these sociologists developed survey instruments that capture Neo-Marxist notions of class (Kohn and Schooler, 1969; Wright and Perrone, 1977; Robinson and Kelley, 1979; Wright, 1979; McNamee and Vanneman, 1987). Among these sociologists, Erik Olin Wright had the major impact in the health sciences during the eighties and beyond (Krieger *et al*, 1997; Muntaner *et al*, 2013). A leading theoretical production during Analytical Marxism's decade and afterwards (Wright, 1985), a long-term commitment to Marxist sociology (Wright, 1994), a number of highly visible empirical articles (Wright, 1997), and a continuous engagement with Weberian sociology (Wright, 1989) could explain such influence.

## Neo-Marxist Class Analysis in Population Health

Although some early articles used Neo-Marxist class indicators to tap into psychological outcomes such as self-esteem (Kohn and Schooler, 1969), it was not until the 1990s that health researchers began using Wright's Neo-Marxist class indicators in population surveys. In brief, Wright's survey questions for the measurement of social class tap into three theoretical constructs of control over productive assets: ownership, organization, and skills/credentials. The construct of skill/credentials is close to that of ‘education', used commonly in the social inequalities in health field. Its status as a Neo-Marxist relational construct has been debated since the publication of *classes* (Wright, 1985) and is generally considered a Neo-Weberian influence on Wright's model (Wright, 1989). Nonetheless, higher skills/credentials can result in higher wages (that is, less exploitation) and more autonomy (that is, less domination) than lower skills/credentials, indicating its theoretical salience for relation to the means of production. In the first version of his model, Wright emphasized the construct of *control* (that is, domination) over production relations (economic ownership, control over the means of production, and control over labor (Wright, 1979). It was not until later when, influenced by John Roemer's rational choice Marxism, he shifted his theoretical focus to the exploitation mechanism (Wright, 1985).

Ownership of productive assets refers to the relations to the means of production (large employer, small employer, petit-bourgeois and worker) and control of organizational assets refers to relations of control (that is, domination) over labor power at the work place (manager, supervisor, non-managerial worker). Indicators of these class relations (Wright, 1997) typically include a question on how many workers the respondent employs (ownership); a question on whether the respondent can hire and fire other workers (organizational control); and a question on whether the respondent has the power to influence company policy (budgets, inventories, and investments). Workers who influence company policy and can fire and fire workers are assigned the class position of manager; those who can only hire or fire are assigned the class position of supervisor; and those who cannot perform any of these functions are assigned the class position of non-managerial workers. With three class locations per dimension, Wright's theoretical Neo-Marxist class framework consists of 12 class positions or locations (Wright, 1997). Another component of Wright's framework is the construct of contradictory class locations (CCL) in production relations. For example, a supervisor is a manager to workers and a worker to managers, a contradictory position that might have consequences for her lived experience at work and class consciousness. This framework and its indicators have been applied to population health problems since the mid-1990s, producing a set of new findings appraised in the next section.

## A Critical Appraisal of the Extant Literature

The amount of existing empirical Neo-Marxist research pales in comparison with their theoretical counterparts. Within the empirical domain, for example, there are far fewer studies using Neo-Marxist conceptual definitions of class than Neo-Weberian (Blane *et al*, 1993) or Bourdieusian measures (Sayer, 2005; Veenstra, 2006 ). In order to gain a better sense of the nature and scope of the Neo-Marxist literature, in February 2015, we searched for empirical, peer-reviewed studies in *PubMed* with the following key word terms in the Title or Abstract: ‘Neo-Marxist', ‘Wright', ‘social class' and ‘contradictory class location'. A total of 19 studies met our conceptual definition of social class and methodological criteria of quantitative designs. As revealed in Table 1, our own research network has contributed eleven of the 19 studies to the Neo-Marxist literature (Borrell *et al*, 2004, 2008; Espelt *et al*, 2008; Muntaner *et al*, 1994, 1998, 2003, 2009, 2015; Muntaner and Parsons, 1996; Rocha *et al*, 2013, 2014; Prins *et al*, in press), thus allowing us to critically appraise the strengths, limitations, and future needs of this scholarship. This list of studies overlaps and updates our previous systematic review on the relational effects of social class on health (Muntaner *et al*, 2010). Together, these studies reflect several common characteristics. First, the vast majority of Neo-Marxist studies have used cross-sectional designs (89.5 per cent, 17 of 19 studies), analysed survey data from the general population (78.9 per cent, 15 of 19 studies), and tested self-rated health and mental health as dependent variables (84.2 per cent, 16 of 19 studies). Second, only 2 of the 19 studies used longitudinal designs (10.5 per cent) (Macleod *et al*, 2005; Muntaner *et al*, 2009), revealing a clear and urgent need for research to repeatedly collect data on the same employers and employees to establish causality. Third, the over-reliance on general population surveys has meant that certain social class positions are more likely to be systematically excluded from observation. This includes social class positions such as larger employers and the most exploited workers, which in turn, narrows and underestimates the true range of effect estimates. And fourth, research has paid scant attention to the instrumental role that Neo-Marxist class plays in shaping and influencing other social determinants of health, including for example, gender, race/ethnicity, disability, national or religious relations, access to services, employment conditions, income and wealth or at different levels of aggregation (for example, firm, neighborhood, and city). One notable exception is Borrell *et al* (2004)'s study that tested multiple pathways such as psychosocial stress, housework, material deprivation through which Neo-Marxist class positions might generate health inequalities among men and women.

Yet Neo-Marxist class indicators are consistently associated with predictable health outcomes, with large and medium size employers and managers showing the healthiest profiles (Schwalbe and Staples, 1986; Muntaner and Parsons, 1996; Wohlfarth, 1997; Wohlfarth and van den Brink, 1998; Muntaner *et al*, 1998, 2003, 2009, 2015; Borrell *et al*, 2004, 2008; Rocha *et al*, 2013, 2014; Hadewijch *et al*, 2014). In particular, studies that conceptualize and test CCL hypotheses demonstrate the added value of Neo-Marxist thinking and analysis. The key advantage is that CCL hypotheses suggest social mechanisms that do not follow the gradient (Muntaner *et al*, 1998; Prins *et al*, in press). That is, gradient hypotheses postulate that supervisors would present worse mental health than managers but better than frontline workers. Here, the idea is that increases in income correspond with improvements in health. In contrast, CCL hypotheses are based on the domination from managers and the opposition of workers to their domination by supervisors. Instead of graded health outcomes, one would anticipate worse mental health among supervisors than among frontline, non-managerial workers (Muntaner *et al*, 1998). Separate findings from the United States, Spain, and Chile suggest that this might be the case among frontline, low-level supervisors as identified by Wright's indicator of social class locations (Muntaner *et al*, 1998; Muntaner *et al*, 2003; Rocha *et al*, 2013; Hadewijch *et al*, 2014, Prins *et al*, in press). Compared with an occupational gradient hypothesis, Neo-Marxist class analysis reveals relational class mechanisms that make a logical link between psychosocial and proximal processes (for example, lack of control, high demands) and different, non-linear predictions (for example, increases in income do not automatically translate into enhanced health) (Muntaner *et al*, 2003). Moreover, the theorized organizational control mechanisms among manager, supervisor, and non-managerial worker relations ground Neo-Marxist class analysis in realist epistemology (Scambler, 2001; Scambler *et al*, 2002).

Overall, these Neo-Marxist studies reveal a genuine lack of correspondence between theoretical definitions and social class measures. For example, Wright's definition of social class postulates that the key mechanism generating inequalities in economic welfare between social class positions is *exploitation*. Exploitation is defined by Wright in Neo-Marxist terms, coupling it with power and domination during production. Three principles are invoked to define this key class mechanism (Wright, 1997):

1. *The inverse interdependent welfare principle*: the material welfare of exploiters causally depends upon the material deprivations of the exploited. This means that the interests of actors within such relations are not merely different, they are antagonistic: the realization of the interests of exploiters imposes harms on the exploited.
2. *The exclusion principle*: this inverse interdependence of the welfare of exploiters and exploited depends upon the exclusion of the exploited from access to certain productive resources.
3. *The appropriation principle*: Exclusion generates material advantage to exploiters because it enables them to appropriate the labor effort of the exploited.

Yet survey questions used in the existing literature often fail to capture exploitation and its three principles (D'Souza *et al*, 2003). Instead, most studies are measuring employment relations (for example, employer, self-employed owner, worker), which makes them practically analogous to Neo-Weberian indicators of social class (Marshall *et al*, 1988). A heuristic solution to this impasse might be to measure the *process of exploitation*, and its associated domination, with its own set of indicators. For example, in a recent study among nursing assistants and their mental health within the context of US nursing homes, exploitation was measured at the organizational level with for-profit status and domination was measured with rating scales answered by key informants in each workplace (Muntaner *et al*, 2015). Results showed strong multilevel effects of indicators of exploitation and domination on worker mental health. A specific focus on the social mechanisms of exploitation and domination (Wright, 1997) that underlie inequalities in welfare between persons in different class positions could hold promise for future Neo-Marxist class analyses. This would actually represent a significant move towards a more ‘classical' Marxist understanding of class (Wolff and Resnick, 1986) without abandoning the distinctive empirical bent of Wright's ‘Analytical Marxism'.

## Conclusions

### Identifying class mechanisms to guide social change and reduce health inequalities

An ostensible goal of all research on the social production of health inequalities is not merely to describe or explain such inequalities, but to effectively reduce them (Muntaner and Lynch, 2002; O'Campo and Dunn, 2011; Muntaner *et al*, 2012b). A Neo-Marxist class approach has implications for the way that researchers think about and engage with efforts to reduce health inequalities, implications that invert the mainstream relationship between research and action. A cursory glance at the conclusion sections of many population health studies reveals an almost rote focus on ‘policy implications' relevant to policymakers. We argue here that, although this mainstream orientation to social class and health inequalities may appear innocuous or politically neutral, it in fact functions in the service of incremental, apolitical, technical changes that are ultimately system-justifying and *status-quo*-reproducing (Chomsky, 1971).

As we described at the outset, the individual attribute approach to social class tracked broader trends in social science theory and research towards reductionism and methodological individualism. This absolves researchers from engaging with social processes and relations, which demand analyses of exploitation, domination, and even employment relations. These intellectual trends, in turn, reflect structural changes in the political economy of academic institutions that produce such knowledge (Muntaner *et al*, 2012a). While a complete discussion of the impact of neo-liberalism on health inequalities research is beyond the scope of this analysis, we contend that such trends conform to political options that often perpetuate inequalities, because they produce knowledge that explicitly avoids the mechanisms that generate social and health inequalities.

What can a Neo-Marxist approach to social and health inequalities add? Aside from doing the opposite of the mainstream approach (that is, re-engaging with analyses of employment relations, exploitation, domination and other class processes), an important contribution of Neo-Marxist class analysis is to break the chain between health inequality research and the ‘policy mystique'. It can do this by flipping its orientation from the top-down to the bottom-up, and rediscovering and engaging with the rich diversity of poor people's and working class social movements whose struggles – *class struggles* – against inequality, including health inequalities, can become a target audience for research and action. Adopting a relational class approach means recognizing – not just politically, but from a pragmatic research design and implementation perspective – that the vast majority of ‘the 99 per cent' are completely alienated from the policy space, both professionally and electorally. Examples of such bottom up class approaches would be the ‘Housing First' program in Canadian cities (van Draanen *et al*, 2013) or public health action research with labour unions in the United States (Malinowski *et al*, 2015). A resurgence of poor, working class, and climate-justice activism, from the international outgrowths of Latin America's left turn and the Arab Spring (Muntaner *et al*, 2011 ) to the anti-austerity movements in the European Union (Tugas, 2014), provides compelling opportunities for researchers to address new, grassroots stakeholders.

Recognizing that the vast majority of the population is on the opposite side of the class struggle than ‘policymakers' does not imply that we should abandon progressive health policy reforms, but it means that we should adopt a more critical, bottom-up perspective towards how policy changes affecting the public's health are ultimately achieved. This is not to say that all researchers of social inequalities in health must become public social scientists (Burawoy, 2005) but it is to say that we cannot consign ourselves, under a thin veil of neutrality, to *de facto* approaching policy from a privileged position of access to elites, that is, from the orientation of serving policymakers. At the very least, we should have a more class-conscious perspective (Burawoy, 2014). Returning to and advancing relational approaches to class may be the only way this will be possible.

## Future Directions

Previous research on the relational effects of social class on health inequalities confirms the explanatory and analytical value of conceptualizing mechanisms of exploitation and domination as health determinants. Our critical reconstruction of two decades of Neo-Marxist scholarship provides an overview of the state-of-evidence and offers several promising directions for future research. First, there is a clear need to advance the conceptualization and measurement of social class in health inequalities scholarship. As revealed in Table 1, existing studies have primarily relied on Wright's typology of class in capitalist societies to understand health inequalities between capitalists, managers, petty bourgeois, and workers. Wright's contributions to our current understanding of social class are indisputable. However, new contributions are needed. Public health scholars run the real risk falling into an intellectual trap if we fail to consider, integrate, and synthesize other concepts into our explanatory frameworks and research methods. For example, most studies make predictions about the health of individuals based on the assumption that capitalists and workers occupy a single class position at one time. Yet, prior work finds that individuals are often simultaneously engaged in multiple class positions that have the effect of generating different amounts of economic resources and creating different kinds of exploitation relations (for example, an individual can be self-employed and a recipient of welfare assistance while occupying a working poor or underclass position) (Muntaner and Stormes, 1996). Other important needs in terms of advancing the conceptualization and measurement of social class include: identifying new social classes that might have emerged because of changes in capital accumulation and employment relations (for example, financial investors, hedge fund managers, technical middle class workers or precarious workers [Standing, 2011]); investigating untested relational and dimensional aspects of social class (for example, health inequalities between different owners such as capitalists (hires 100 or more employees) versus small employees (hires 2–99) employees versus petit bourgeoisie (hires no more than 1 employee) (Muntaner *et al*, 1999); integrating other major social class (non-stratification) approaches in public health (for example, synthesizing Weberian and Bourdieusian ideas about social class with Neo-Marxism (Bartley *et al*, 1999; Veenstra, 2007); and striking a balance between the theory-method nexus (for example, conceptualizing exploitation at organizational levels with the use of multi-level modelling, Muntaner *et al*, 2015).

Second, future work will benefit from incorporating mechanism-based explanations on how and why social class relations generate avoidable and unfair health inequalities, and under what circumstances. On one hand, existing social class studies have successfully identified plausible mechanisms that may contribute to health inequalities. Exploitation, for example, generates and reproduces health inequalities by (i) ensuring that the material welfare of capitalists comes at the expense of the working class; (ii) excluding the working class from owning productive resources, and (iii) appropriating the labour of the working class in the form of profits for the capitalist class. On the other hand, more theory-driven work is needed that considers how social class mechanisms interact with specific contexts to generate intended and unintended outcomes (for example, income and wealth as well as health outcomes). One such approach is scientific realism, which provides a compelling rationale to unpack context-mechanism-outcome (CMO) patterns (Molnar *et al*, 2015; O'Campo *et al*, 2015). In particular, this approach provides useful analytical tools to compare ‘how social class relations are theorized to generate health inequalities' (for example, exploitation) to ‘rigorous evidence on how social class relations actually generate health inequalities in different situations'. By identifying and testing CMO patterns, researchers will be well-positioned to describe and understand the various contingencies that shape and influence the likelihood that social class relations may generate social, economic, and health outcomes. In turn, such findings can potentially inform how social class relations may be restructured in egalitarian policies (for example, increasing workplace democracy) that most likely trigger causal mechanisms (for example, increasing social solidarity) that narrow health inequalities (for example, among capitalist, managers, and workers) (Ng and Muntaner, 2014).

Third, health sociologists and social epidemiologists can significantly advance the ultimate goal of reducing health inequalities by investigating the egalitarian effects of social change. Instead of reinforcing the *status quo* and asking descriptive questions such as ‘What is the extent of social class inequalities in health?', researchers can make major strides toward changing unequal power relations and narrowing health inequalities by raising moral and political questions (Sayer, 2005). Rather than trying to understand health inequalities in terms of individual attributes and material conditions, future work needs to shed a critical light on how health inequalities are generated and re-produced over time and place by asking ‘Should social class relations remain in their present form?' This kind of public sociology (Burawoy, 2005) enables us to generate a host of new and important queries to be considered, including for example, ‘Should we repeal legal rules that give people and firms effective control over productive means and resources?' ‘How can we democratically un-structure mechanisms of domination and exploitation that allow a small number of owners power over the lives and activities of a large number of workers?' ‘How can we initiate and support social struggles that effectively modify existing power relations, which subsequently reduce the distance between exploiting and exploited class positions?' ‘How will taking action on Neo-Marxist social class locations shape and influence social determinants of health?' and ‘How would such egalitarian changes affect the well-being and health of the population?' The past two decades of research on social class and health inequalities have explored and confirmed the existence and extent of the problem. Over the next two decades, it is essential that we overcome barriers to knowledge production and translation in order to make real progress toward changing and transforming the nature of social class inequalities in health (Muntaner *et al*, 2012a).

## Figures and Tables

**Table 1:** Description of Neo-Marxist studies

*Author, Year*	*Location*	*Design/Sample*	*Neo-Marxist social class measure*		*Health outcome(s)*	*Primary findings*
			*Conceptual definition*	*# of positions*		
Borrell *et al*, 2004	Barcelona, Catalonia, Spain	Cross-sectional Survey, *N*=10 000, aged 16–64 (stratified by gender)	Wright's social class locations	12	Self-rated health	In models adjusting for work organization, household material standards, and household labour: • Among men, the prevalence of poor health is higher among small employers and petit bourgeois, supervisors, semi-skilled and unskilled workers. • Among women, only unskilled workers have poorer health than managers and skilled supervisors (reference category).
Borrell *et al*, 2008	Barcelona, Catalonia, Spain	Cross-sectional Survey, *N*=10 000, aged 16–64 (stratified by gender)	Wright's social class locations	12	Self-rated health	In models adjusting for migration status: • Among men, the prevalence of poor health is higher among small employers, supervisors semi-skilled, supervisors unskilled, semi-skilled workers and unskilled workers than managers, supervisor experts (reference category). • Among women, there are no significant health differences between social class locations.
Hadewijch *et al*, 2014	19 European Union states	Pooled cross-sectional Survey, *N*=28 747, aged 15–64 (stratified by gender)	Wright's social class locations	7	WHO Well-Being Index	In models adjusting for age, psychosocial work environment, employment conditions and relations: • Among men, supervisors and managers with an expert skill level reported a worse mental well-being than supervisors and managers with lower skill levels. • Among women, expert-level supervisors also reported a worse mental well-being than lower-skilled supervisors.
De Moortel *et al*, 2015	21 European Union states	Cross-sectional Survey, *N*=14 107, aged 15–64 (stratified by gender and welfare regime)	Wright's social class locations	7	WHO Well-Being Index	In unadjusted models, prevalence ratios show: • In Basic security/market-oriented welfare regimes female unskilled workers, semiskilled supervisors and expert managers had worse mental well-being than their male counterparts. • Female semi-skilled supervisors and unskilled workers reported worse mental well-being than their male counterparts in Contradictory and Southern welfare regimes respectively.
Espelt *et al*, 2008	9 European countries	Cross-sectional survey, *N*=16 901, aged 50–74 (stratified by gender and political tradition)	Modified version of Wright's social class locations (ownership, education, management)	6	Self-rated health and long-term illness	In models adjusting for social class, age, and employment status: • Men and women who are workers, with low education, and with no power over personnel consistently reported poorer health than men and women are owners, with high education, and with power over personnel.
Hirokawa *et al*, 2009	Japan	Cohort study, *N*=3424, aged 65 and younger (stratified by gender and occupational category)	Occupational position (manager, non-manager)	2	Plasma fibrinogen	In models adjusting for socio-demographics and health behaviours: • Among men, no significant association was found between occupational position and plasma fibrinogen. • Among white-collar women, those in non-managerial positions showed higher levels of fibrinogen than those in managerial positions.
Macleod *et al*, 2005	Scotland, UK	Prospective observational study, *N* =5232 Scottish men, aged 35–64, followed up for 25 years	Workplace status (employee, foreman, manager)	3	All-cause and cause-specific mortality and psychiatric hospitalization	In models adjusting for socio-demographic, risk factors, stress, job satisfaction, and social position variables: • Compared to foremen, employees had a small and imprecisely estimated increased risk of all cause mortality, whereas managers had a more marked decreased risk.
Muntaner *et al*, 1994	Baltimore	Cohort study, *N*=248 white male inpatients, aged 16 and over	Wright's social class locations (expert, marginal, uncredentialed)	3	Type of psychotic disorder and first admission to state mental hospital	In models adjusting for age and diagnosis: • Patients with low levels of skills/credentials are more likely than patients with higher levels of skills/credentials to be admitted to state psychiatric hospitals.
Muntaner and Parsons, 1996	Baltimore	Cross-sectional survey, *N*=397 white respondents, aged 21–89 (male and female)	Wright's social class locations (ownership assets, organizational assets, skills/credential assets)	6	Private health insurance	In models adjusting for age, gender, and marital status: • Owners and managers are more than twice as likely to benefit from a private health insurance plan as are wage earners and non-management workers.
Muntaner *et al*, 1998	East Baltimore	Follow-up interview, *N*=1920 (male and female)	Wright's social class locations (managers, supervisors, workers)	3	Depression, anxiety, alcohol and drug abuse or dependence	In models adjusting for socio-demographics and social class measures: • Lower level supervisors presented higher rates of depression and anxiety disorders than higher level managers.
Muntaner *et al*, 2003	Barcelona, Catalonia, Spain	Cross-sectional survey, *N*=4219, aged 16–64 (stratified by gender)	Wright's social class locations	12	Self-rated health and mental health	In models adjusting for social stratification, education, and age: • Among men, high level managers and supervisors reported better health than all other classes. • Low-level supervisors reported worse mental health than high-level managers and non-managerial workers. • Social class indicators were less useful correlates of health and mental health among women.
Muntaner *et al*, 2009	Barcelona, Catalonia, Spain	Longitudinal survey, *N*=7526 (stratified by gender)	Wright's social class locations	6	All-cause mortality	• Among men, mortality rates are significantly higher among unskilled workers compared with capitalists. • Among women, social class is not predictive of mortality rates.
Muntaner *et al*, 2015	Kentucky, Ohio, West Virginia	Cross-sectional survey, *N*=868 (male and female)	Social class exploitation (organizational level)	3	Depressive symptoms	Using two-level models adjusting for age, race, and marital status: • Private for-profit ownership and higher managerial domination are predictive of depression among nursing assistants.
Prins *et al*, in press	USA	Cross-sectional survey, *N*=21 859	Wright's social class locations	4	DSM-IV diagnostic criteria for depression and anxiety	• Occupants of contradictory class locations have higher prevalence and odds of depression and anxiety than occupants of non-contradictory class locations
Rocha *et al*, 2013	Chile	Cross-sectional survey, *N*=9503 (stratified by gender)	Wright's social class locations	13	Self-rated health and mental health, health-related behaviour	• Medium employers have a lower prevalence of poor health. • Unskilled managers have the lowest mental health risk. • Large employers smoke the least, while large employers, expert supervisors, and semi-skilled workers engage in significantly more physical activity.
Rocha *et al*, 2014	Chile	Cross-sectional survey, *N*=9503 (stratified by gender)	Wright's social class locations	7	Self-rated health and mental health	• Inequalities exist in the distribution of psychosocial occupational risk factors by social class. • Neo-Marxist social classes are associated with unequal distributions of self-rated health and mental health.
Schwalbe and Staples, 1986	California	Cross-sectional survey, *N*=253 (male and female)	Wright's social class locations	4	Self-esteem and stress	• Working-class positions are subject to greater routinization and less control. • Routinization and control affect health through the psychological consequences of the immediate work experience it engenders (for example, self-esteem and stress).
Veenstra, 2006	British Columbia	Cross-sectional survey, *N*=655, aged 18–64	Neo-Marxist measure of social class	8	Morbidity, mental health, and self-rated health	Using zero-order tests, class position scheme was not significantly associated with injuries, illnesses, body-mass index, mental health and self-rated health.
Wohlfarth, 1997	Israel	Cross-sectional survey, *N*=2741, aged 24–33 (stratified by gender)	Neo-Marxist measure of social class	8	Psychopathology (PERI symptom scales and RDC diagnoses)	• Neo-Marxist social class measures are able to predict psychopathology, particularly with diagnoses of drug use disorders and depression and with symptom scales of antisocial history, demoralization, enervation, suicide and schizoid traits that cannot be accounted for by SES measures.
Wohlfarth and van den Brink, 1998	Israel	Cross-sectional survey, *N*=2741, aged 24–33 (stratified by gender and ethnicity)	Neo-Marxist measure of social class	8	Substance use disorders (SUDs)	• Advantaged social classes in terms of ownership, that is, self-employed, have higher rates of SUDs compared with employees. • Most disorders have an onset subsequent to entry into the current job, indicating that ownership plays a causal role in the onset of SUDs rather than the other way around.
